# Alternating proximal penalization algorithm for the modified multiple-sets split feasibility problems

**DOI:** 10.1186/s13660-018-1641-y

**Published:** 2018-02-20

**Authors:** Xueyong Wang

**Affiliations:** School of Management, Qufu Normal University, Shandong, China

**Keywords:** 90C25, 94A08, Multiple-sets feasibility problem, Alternating direction method, Penalty method, Convergence

## Abstract

In this paper, we present an extended alternating proximal penalization algorithm for the modified multiple-sets feasibility problem. For this method, we first show that the sequences generated by the algorithm are summable, which guarantees that the distance between two adjacent iterates converges to zero, and then we establish the global convergence of the algorithm provided that the penalty parameter tends to zero.

## Introduction

The multiple-sets split feasibility problem, abbreviated as MSFP, is to find a point closest to the intersection of a family of some closed and convex sets in one space, such that its image under a linear operator is closest to the intersection of another family of some closed and convex sets in the image space. More specifically, the MSFP consists in finding a point $x^{*}$ such that

1.1 $$ x^{*} \in \bigcap_{i=1}^{s} C_{i} \quad \text{and} \quad Ax^{*} \in \bigcap ^{t}_{j=1} Q_{j}, $$

where $C_{i} \subset R^{m}$ ($i=1,2,\ldots, s$) and $Q_{j} \subset R ^{n} $ ($j=1,2,\ldots, t$) are nonempty, closed, and convex sets in a Euclidean space, $A \in R^{n \times m}$ is a given matrix.

The problem finds applications in such fields as image reconstruction and signal processing [1], and it was intensively considered by researchers [2–11]. For this problem, numerous efficient iterative methods were proposed, see [12–23] and the references therein.

To solve the problem, based on the multidistance idea, Censor and Elfving [1] established an iterative algorithm which involves the inverse of the underlying matrix at each iteration and hence is much more time-consuming. To overcome this drawback, Byrne [24] presented a projection-type algorithm, called CQ method, for solving the split feasibility problem. The algorithm is efficient when the orthogonal projections can be easily calculated. To make the projection method more efficient when the projection is difficult to compute, Yang [25] established a relaxed CQ algorithm by modifying the projection region. For this method, to make the objection function have sufficient decrease at each iteration, Qu and Xiu [26] presented a revised CQ method by introducing an Armijo-like stepsize rule into the iterative frame. In order to accelerate the speed of the algorithm, Zhang and Wang [27] made a further modification to the method by adopting a new searching direction for the split feasibility problem.

Inspired by the work in [1] and the alternating proximal penalization algorithm in [28], we present a two-block alternating proximal penalization algorithm for this problem in this paper. Under mild conditions, we first show that the sequences generated by our algorithm are summable, which guarantees that the distance between two adjacent iterates converges to zero, and then we establish the global convergence of the algorithm provided that the penalty parameter tends to zero.

The remainder of this paper is organized as follows. In Sect. 2, we give some basic definitions and lemmas which will be used in the subsequent sections. In Sect. 3, we present a new method for solving the split problem and establish its convergence. Some conclusions are drawn in the last section.

## Preliminaries

In this section, we first present some definitions and then recall some existing conclusions which will be used in the subsequent analysis.

First, we give some definitions on the continuous function $f: R^{n} \to R$.

### Definition 2.1

([29])

Let $f:\Omega (\subset R^{n}) \to \Omega $ be a continuous function. Then

1. *f* is called monotone on Ω if
  $$ ( u-v)^{T} \bigl(f(u)-f(v) \bigr) \ge 0, \quad \forall u ,v \in \Omega ; $$
2. *f* is called *ν*-inverse strongly monotone on Ω if there exists a constant $\nu > 0$ such that
  $$ ( u-v)^{T} \bigl(f(u)-f(v) \bigr)\ge \nu \bigl\Vert f(u)-f(v) \bigr\Vert ^{2} , \quad \forall u ,v \in \Omega ; $$
3. *f* is called Lipschitz continuous on Ω if there exists a constant $L > 0$ such that
  $$ \bigl\Vert f(u)-f(v) \bigr\Vert \le L \Vert u-v \Vert , \quad \forall u, v \in \Omega ; $$
4. The subgradient set of *f* at *x* is given by
  $$ \partial f(x):= \bigl\{ \xi \in R^{n} : f(y) \ge f(x) + \xi^{T} (y-x ), \forall y \in R^{n} \bigr\} . $$

### Lemma 2.1

([30])

*For functions* $f(x)= \frac{1}{2} \sum_{i = 1}^{s} a_{i} \Vert x -P _{C_{i}}(x) \Vert ^{2}$ *and* $g(y)=\frac{1}{2} \sum_{j=1}^{t} b_{j} \Vert y -P_{Q_{j}}(y) \Vert ^{2}$, *it holds that* $\nabla f(x)$ *and* $\nabla g(y)$ *are both inverse strongly monotone and Lipschitz continuous on X and Y*, *where* $P_{C_{i}}(x)$ *denotes the projection of* *x* *onto* $C_{i}$, *i*.*e*., $P_{C_{i}}(x)=\arg \min \{ \Vert y-x \Vert \mid y \in C_{i}\}$.

To proceed, we give some conclusions which play the heart role in the next section.

### Lemma 2.2

[28] *Let* $\{a_{n} \}$ *and* $\{ \epsilon_{n} \}$ *be two real sequences such that* $\{a_{n} \}$ *is minorized*, $\{ \epsilon_{n} \} \in l^{1}$, *and* $a_{n+1} \le a_{n} + \epsilon_{n} $ *for any* $n \in N$. *Then sequence* $\{a_{n}\}$ *converges*.

### Lemma 2.3

(Opial lemma [31])

*Let* $\{\lambda_{k} \} $ *be a nonsummable sequence of positive real numbers*, *and* $\{ x^{k} \}$ *be any sequence in a Hilbert space* *H*, *with the weighted averages* $\{ z ^{k} \} $. *Assume that there exists a nonempty closed convex subset* *F* *of* *H* *such that*

1. *weak subsequential limits of* $\{ z^{k} \}$ *lie in* *F*,
2. $\lim_{k \to \infty } \Vert x^{k} - f \Vert $ *exists for all* $f \in F $.

*Then* $\{ z ^{k} \} $ *converges weakly to an element of F*.

To end this section, we define the following operators which will be used in the following sections.

Let $V:= \{(x,y) : x \in X, y \in Y, Ax=y \}$. Define monotone operators *F̂* and *Ĝ* as follows:

$$\begin{aligned}& \hat{F}(x,y)= \bigl(f(x),g(y) \bigr), \\& \hat{G}(x,y)= \partial \hat{F}(x,y)+N_{V}(x,y)= \textstyle\begin{cases} \partial \hat{F}(x,y)+V^{\bot },&\text{if } (x,y) \in V , \\ \emptyset ,& \text{if } (x,y) \notin V. \end{cases}\displaystyle \end{aligned}$$

Further, we define bounded linear operators ϒ and Ψ as follows:

$$ \Upsilon : \quad \begin{gathered} X \times Y \mapsto Z , \\ (x,y) \mapsto Ax-y, \end{gathered}\quad\text{and}\quad \Psi :\quad \begin{gathered} X \times Y \mapsto R, \\ (x,y) \mapsto \frac{1}{2} \Vert Ax-y \Vert ^{2}. \end{gathered} $$

Let Ω be a nonempty closed convex subset of $R^{n}$. Then the normal cone operator of Ω at *x* is defined as

$$ N_{\Omega }(x) := \bigl\{ x^{*} : \bigl( x^{*} \bigr)^{T} (y-x ) \le 0, \forall y \in \Omega \bigr\} . $$

For the function $\Psi : X \times Y \mapsto R $, the Fenchel conjugate $\Psi^{*} $ of the map Ψ at $p \in X \times Y $ is given by

$$ \Psi^{*} (p): = \sup \bigl\{ p^{T} q - \Psi (q) : q \in X \times Y \bigr\} . $$

## Algorithm and the convergence analysis

In [30], Zhang *et al.* proposed an alternating direction method to solve problem (1.1) based on the Lagrange function. Different from it, in this paper, we propose the following alternating proximal penalization algorithm: Given the current iterate $(x^{k},y ^{k})$ and positive parameters *α* and *β*, the new point $(x^{k+1},y^{k+1})$ is generated by

3.1 $$ \begin{gathered} x^{k+1}= \operatorname{argmin} \biggl\{ \gamma_{k+1}f(x)+ \frac{1}{2} \bigl\Vert Ax-y^{k} \bigr\Vert ^{2} + \frac{\alpha }{2} \bigl\Vert x -x^{k} \bigr\Vert ^{2} : x \in X \biggr\} , \\ y^{k+1}= \operatorname{argmin} \biggl\{ \gamma_{k+1}g(y)+ \frac{1}{2} \bigl\Vert Ax^{k+1}-y \bigr\Vert ^{2} + \frac{\beta }{2} \bigl\Vert y -y^{k} \bigr\Vert ^{2} : y \in Y \biggr\} . \end{gathered} $$

Note that the penalty parameter sequence satisfies $\{ \gamma_{k} \} \in l^{2}/l^{1}$.

In order to investigate the convergence of the algorithm, we define the following estimation function:

$$ h_{k}(x,y)= \alpha \bigl\Vert x^{k} -x \bigr\Vert ^{2} +(\beta +1) \bigl\Vert y^{k}-y \bigr\Vert ^{2} $$

for $(x,y) \in X \times Y $.

### Lemma 3.1

*Let* $(x,y) \in X \times Y$ *and* $(\xi , \eta ) \in \hat{G}(x,y)$. *Then there exists* $(p,q) \in (X \times Y)^{\bot }$ *such that*

3.2 $$\begin{aligned} \begin{aligned}[b] & h_{k+1}(x,y) - h_{k}(x,y) + 2 \gamma_{k+1} \bigl[ \xi^{T} \bigl(x^{k+1}-x \bigr) + \eta^{T} \bigl(y^{k+1}-y \bigr) \bigr] \\ &\quad{} +\alpha \bigl\Vert x^{k+1}-x^{k} \bigr\Vert ^{2} + \beta \bigl\Vert y^{k+1}- y^{k} \bigr\Vert ^{2}+ \bigl\Vert Ax^{k+1}-y^{k} \bigr\Vert ^{2} \le 2 \gamma_{k+1 }^{2} \Psi^{*}(p,q). \end{aligned} \end{aligned}$$

### Proof

From (3.1), one has

3.3 $$ \frac{\alpha }{\gamma_{k+1}} \bigl(x^{k+1}-x^{k} \bigr) + \frac{1}{\gamma_{k+1}}A ^{T} \bigl(Ax^{k+1}-y^{k} \bigr) = - \nabla f \bigl(x^{k+1} \bigr). $$

On the other hand, since $(\xi , \eta ) \in \hat{G}(x,y)$, there exists $(p,q) \in (X \times Y)^{\bot }$ such that

$$ \xi = \nabla f(x) + p \quad \text{and} \quad \eta = \nabla g(y) + q , $$

which implies that $p - \xi = -\nabla f(x)$. Combining this with (3.3) and using the monotonicity of $\nabla f(x)$, one as

$$ \frac{\alpha }{\gamma_{k+1}} \bigl( x ^{k+1} - x^{k} \bigr)^{T} \bigl(x ^{k+1} - x \bigr) + \frac{1}{\gamma_{k+1}} \bigl( A^{T} \bigl(Ax^{k+1}-y^{k} \bigr) \bigr)^{T} \bigl( x^{k+1}-x \bigr) \le ( p-\xi )^{T} \bigl( x^{k+1}-x \bigr). $$

Rearranging the item of the inequality yields

3.4 $$ \begin{aligned}[b] \alpha \bigl\Vert x^{k+1}-x \bigr\Vert ^{2} + \alpha \bigl\Vert x^{k+1}-x^{k} \bigr\Vert ^{2} &\le \alpha \bigl\Vert x^{k}-x \bigr\Vert ^{2} -2 \bigl( Ax^{k+1}-y^{k} \bigr)^{T}\bigl(Ax^{k+1}-Ax \bigr) \\ &\quad{} +2 \gamma_{k+1} p^{T} \bigl(x^{k+1}-x \bigr)- 2\gamma_{k+1} \xi^{T}\bigl( x^{k+1}-x \bigr). \end{aligned} $$

Similarly, one has

3.5 $$ \begin{aligned}[b] \beta \bigl\Vert y^{k+1}-y \bigr\Vert ^{2} + \beta \bigl\Vert y^{k+1}-y^{k} \bigr\Vert ^{2} &\le \beta \bigl\Vert y^{k}-y \bigr\Vert ^{2} -2 \bigl( y^{k+1}-Ax^{k+1} \bigr)^{T}\bigl(y^{k+1} -y \bigr) \\ &\quad{} +2 \gamma_{k+1} q^{T}\bigl(y^{k+1}-y \bigr)- 2\gamma_{k+1} \eta^{T} \bigl( y^{k+1}-y \bigr). \end{aligned} $$

Note that $Ax =y $ implies

$$\begin{aligned}& 2 \bigl( Ax^{k+1}-y^{k} \bigr)^{T} \bigl( Ax^{k+1}-Ax \bigr)+2 \bigl( y^{k+1} -Ax^{k+1} \bigr)^{T} \bigl(y ^{k+1} -y \bigr) \\& \quad = \bigl\Vert y^{k+1}-y \bigr\Vert ^{2} + \bigl\Vert Ax^{k+1} -y^{k} \bigr\Vert ^{2} + \bigl\Vert Ax^{k+1}- y^{k+1} \bigr\Vert ^{2} - \bigl\Vert y^{k} - y \bigr\Vert ^{2}. \end{aligned}$$

Then (3.4) and (3.5) yield

3.6 $$\begin{aligned}& h_{k+1}(x,y) - h_{k}(x,y) + 2 \gamma_{k+1} \bigl[ \xi^{T} \bigl(x^{k+1}-x \bigr) + \eta^{T} \bigl( y^{k+1}-y \bigr) \bigr] +\alpha \bigl\Vert x^{k+1}-x^{k} \bigr\Vert ^{2} \\& \quad\quad{} + \beta \bigl\Vert y^{k+1}- y^{k} \bigr\Vert ^{2} + \bigl\Vert Ax^{k+1}-y^{k} \bigr\Vert ^{2} \\& \quad \le 2\gamma_{k+1}(p,q)^{T} \bigl(x^{k+1},y^{k+1} \bigr) - \bigl\Vert Ax^{k+1}-y^{k+1} \bigr\Vert ^{2} \\& \quad = 2 \bigl[\gamma_{k+1}(p,q)^{T} \bigl(x^{k+1},y^{k+1} \bigr) - \Psi \bigl(x^{k+1},y^{k+1} \bigr) \bigr] \\& \quad \le 2 \sup \bigl\{ \gamma_{k+1}(p,q)^{T} \bigl(x^{k+1},y^{k+1} \bigr) - \Psi \bigl(x^{k+1},y ^{k+1} \bigr) \bigr\} , \end{aligned}$$

where we use the fact that

$$ p^{T} \bigl(x^{k+1}-x \bigr) + q^{T} \bigl(y^{k+1}-y \bigr) = p^{T} \bigl( x^{k+1} \bigr) + q^{T} \bigl( y ^{k+1} \bigr) =(p,q)^{T} \bigl(x^{k+1},y^{k+1} \bigr) $$

for $(x,y) \in X \times Y$ and $(p,q) \in (X \times Y)^{\bot }$.

By the definition of $\Psi^{*}$, one has

$$ \sup \bigl\{ \gamma_{k+1}(p,q)^{T} \bigl(x^{k+1},y^{k+1} \bigr) - \Psi \bigl(x^{k+1},y^{k+1} \bigr) \bigr\} = \Psi^{*} \bigl(\gamma_{k+1}(p,q) \bigr)= \gamma_{k+1}^{2} \Psi^{*}(p,q). $$

Substituting it into (3.6), we obtain inequality (3.2) and this completes the proof. □

Since the closeness of $R(\Upsilon )$ is equivalent to the closeness of $R(\Upsilon^{*})$, one has $(X \times Y)^{\bot }=\operatorname{Ker} ( \Upsilon )^{\bot } = R (\Upsilon^{*})$, which means that $(X \times Y)^{\bot } \subset \operatorname{dom}\Psi^{*} = R(\Upsilon^{*})$. Thus, $\Psi^{*}(p,q) < +\infty $, $\forall (p,q) \in (X \times Y) ^{\bot } $.

### Lemma 3.2

*For the function* ϒ *defined at the end of Sect*. 2 *and* $(x^{*}, y^{*}) \in \Omega $, *then*

1. $\lim_{k \to +\infty } h_{k} (x^{*}, y^{*}) $ *exists*, *and the sequence* $\{ (x^{k},y^{k}) \} $ *is bounded*;
2. *sequences* $\{ \Vert x^{k+1}-x^{k} \Vert ^{2} \}$, $\{ \Vert y^{k+1}-y ^{k} \Vert ^{2} \}$, *and* $\{ \Vert Ax^{k }-y^{k} \Vert ^{2} \}$ *are summable*.

*In particular*,

$$ \lim_{k \to +\infty } \bigl\Vert x^{k+1}-x^{k} \bigr\Vert = \lim_{k \to +\infty } \bigl\Vert y^{k+1}-y^{k} \bigr\Vert = \lim_{k \to + \infty } \bigl\Vert Ax^{k }-y^{k} \bigr\Vert =0, $$

*and each cluster point of the sequence* $\{ (x^{k}, y^{k} ) \} $ *lies in* $X \times Y$.

### Proof

(1) Setting $(\xi , \eta ) = (0,0)$ in (3.2) and taking $h_{k} = h_{k}(x^{*}, y^{*})$, one has

3.7 $$\begin{aligned} h_{k+1}- h_{k} + \alpha \bigl\Vert x^{k+1}-x^{k} \bigr\Vert ^{2} + \beta \bigl\Vert y^{k+1}- y ^{k} \bigr\Vert ^{2} + \bigl\Vert Ax^{k+1}-y^{k} \bigr\Vert ^{2} \le 2 \gamma_{k+1 }^{2} \Psi ^{*}(p,q). \end{aligned}$$

Hence, $h_{k+1}- h_{k} \le 2 \gamma_{k+1 }^{2} \Psi^{*}(p,q)$. The closedness of $R (\Upsilon )$ and Lemma 2.2 imply that the sequence $\{ h_{k} \}$ converges, which means that $\lim_{k \to +\infty } h_{k} (x^{*}, y^{*})$ exists and the sequence $\{ (x^{k},y^{k}) \} $ is bounded.

(2) Summing inequality (3.7) for $k= 1,2,\ldots,n $, one has

3.8 $$\begin{aligned} &h_{n+1}- h_{1} + \alpha \sum _{k=1}^{n} \bigl\Vert x^{k+1}-x^{k} \bigr\Vert ^{2} + \beta \sum_{k=1}^{n} \bigl\Vert y^{k+1}- y^{k} \bigr\Vert ^{2} + \sum_{k=1}^{n} \bigl\Vert Ax^{k+1}-y^{k} \bigr\Vert ^{2} \\ &\quad \le 2 \sum_{k=1}^{n} \gamma_{k+1 }^{2} \Psi^{*}(p,q). \end{aligned}$$

Since $R (\Upsilon )$ is closed, passing onto the limit on both sides of (3.8), one has that sequences

$$ \bigl\{ \bigl\Vert x^{k+1}-x^{k} \bigr\Vert ^{2} \bigr\} , \quad\quad \bigl\{ \bigl\Vert y^{k+1}-y^{k} \bigr\Vert ^{2} \bigr\} , \quad \text{and} \quad \bigl\{ \bigl\Vert Ax^{k+1 }-y^{k} \bigr\Vert ^{2} \bigr\} $$

are all summable.

Since $\Vert Ax^{k} -y^{k} \Vert ^{2} \le 2 \Vert Ax^{k+1}-y^{k} \Vert ^{2}+ 2 \Vert A x ^{k+1}-Ax^{k} \Vert ^{2}$, $\{ \Vert Ax^{k} -y^{k} \Vert ^{2} \}$ is summable. Further, since the function Ψ is weak lower-semicontinuous, $\lim_{k\to + \infty } \Vert Ax^{k} -y^{k} \Vert = 0$ implies that every cluster point of the sequence $\{ (x^{k}, y^{k} ) \} $ lies in $X \times Y$. □

In order to prove the convergence of the algorithm, we need the following notations.

Setting $\tau_{k} = \sum_{n=1}^{k} \gamma_{n}$, define the averages of the sequences $\{x^{k} \}$ and $\{ y^{k} \}$ as

$$ \hat{x}^{k} = \frac{1}{\tau_{k}} \sum_{n=1}^{k} \gamma_{n} x ^{n} \quad \text{and} \quad \hat{y}^{k} = \frac{1}{\tau_{k}} \sum_{n=1} ^{k} \gamma_{n} y ^{n} . $$

Now we are in a position to prove the convergence of the algorithm.

### Theorem 3.1

*Let the space* $R (\Upsilon )$ *be closed and* Ω *be nonempty*. *Then the sequence* $\{ (\hat{x}^{k},\hat{y}^{k}) \}$ *defined above converges to a point in *Ω.

### Proof

We break up the proof into two parts.

First, we prove that every cluster point of the sequence $\{ (\hat{x} ^{k},\hat{y}^{k}) \}$ lies in Ω.

In fact, for any $(\xi , \eta ) \in \hat{G}(x,y)$, summing inequality (3.2) in Lemma 3.1 for $k =0,1,\ldots, {n-1}$, one has

3.9 $$\begin{aligned} \xi^{T} \bigl(\hat{x}^{k}-x \bigr) + \eta^{T} \bigl(\hat{y}^{k} -y \bigr) \le \frac{1}{2\tau _{k}} \Biggl[ h_{0}(x,y)+2\Psi^{*}(p,q) \sum _{n=1}^{k} \gamma_{n} ^{2} \Biggr]. \end{aligned}$$

Let $(\hat{x}^{*},\hat{y}^{*}) $ be a cluster point of $\{(\hat{x} ^{k},\hat{y}^{k}) \}$. Since $R (\Upsilon )$ is closed, passing onto the limit on both sides of (3.9), one has

$$ \xi^{T} \bigl( \hat{x}^{*}-x \bigr) + \eta^{T} \bigl( \hat{y}^{*}-y \bigr) \le 0, $$

where we use the fact that $\lim_{k \to + \infty } \sum_{n=1}^{k} \gamma_{n}^{2}$ exists, and $\tau_{k} =\sum_{n=1}^{k} \gamma_{n} \to +\infty $ as $n \to +\infty $. From the arbitrariness of $(\xi , \eta )$, one obtains that $(\hat{x}^{*}, \hat{y}^{*}) \in \Omega $.

Second, we prove that the sequence $\{ (\hat{x}^{k},\hat{y}^{k}) \}$ has at most one cluster point.

Let $(\hat{x}_{1}^{*},\hat{y}_{1}^{*}) \ne (\hat{x}_{2}^{*},\hat{y} _{2}^{*}) $ be the two cluster points of the sequence $\{ (\hat{x} ^{k},\hat{y}^{k}) \}$. Define the function

$$ H(u,v)= \alpha \Vert u \Vert ^{2}+ \beta \Vert v \Vert ^{2}, \quad \forall (u,v) \in X \times Y. $$

From (1) in Lemma 3.2, one has that $\lim_{k \to \infty } H( \hat{x}^{k}-x^{*}_{1}, \hat{y}^{k}-y^{*}_{1})$ and $\lim_{k \to \infty } H(\hat{x}^{k}-x^{*}_{2}, \hat{y}^{k}-y^{*} _{2})$ exist.

On the other hand, since

$$\begin{aligned} H \bigl(\hat{x}^{k}-x^{*}_{1}, \hat{y}^{k}-y^{*}_{1} \bigr)&= H \bigl( \hat{x}^{k}-x ^{*}_{2}, \hat{y}^{k}-y^{*}_{2} \bigr) + H \bigl(x^{*}_{1}-x^{*}_{2}, y^{*}_{1}-y ^{*}_{2} \bigr) \\ &\quad{} +2 \alpha \bigl\langle \hat{x}^{k}- x^{*}_{2} ,x^{*}_{2} - x ^{*}_{1} \bigr\rangle + 2 \beta \bigl\langle \hat{y}^{k}- y^{*}_{2} ,y^{*}_{2} - y ^{*} _{1} \bigr\rangle , \end{aligned}$$

the definition of $(\hat{x}_{2}^{*},\hat{y}_{2}^{*}) $ yields

3.10 $$ \lim_{k \to \infty } H \bigl(\hat{x}^{k}-x^{*}_{1}, \hat{y}^{k}-y ^{*}_{1} \bigr) = \lim _{k \to \infty } H \bigl(\hat{x}^{k}-x^{*}_{2}, \hat{y}^{k}-y^{*}_{2} \bigr)+ H \bigl(x^{*}_{1}-x^{*}_{2}, y^{*}_{1}-y^{*}_{2} \bigr). $$

For the same reason, one has

3.11 $$ \lim_{k \to \infty } H \bigl(\hat{x}^{k}-x^{*}_{2}, \hat{y}^{k}-y ^{*}_{2} \bigr)= \lim _{k \to \infty } H \bigl(\hat{x}^{k}-x^{*}_{1}, \hat{y}^{k}-y^{*}_{1} \bigr)+ H \bigl(x^{*}_{1}-x^{*}_{2}, y^{*}_{1}-y^{*}_{2} \bigr). $$

Then (3.10) and (3.11) imply that $H(x^{*}_{1}-x^{*}_{2}, y^{*}_{1}-y^{*}_{2})=0 $, which means that $(\hat{x}_{1}^{*},\hat{y} _{1}^{*}) = (\hat{x}_{2}^{*},\hat{y}_{2}^{*}) $. This contradicts $(\hat{x}_{1}^{*},\hat{y}_{1}^{*}) \ne (\hat{x}_{2}^{*},\hat{y}_{2} ^{*})$, and thus the sequence $\{ (\hat{x}^{k},\hat{y}^{k}) \}$ has at most one cluster point and the proof is completed. □

## Conclusion

In this paper, we presented an extended alternating proximal penalization algorithm for the modified multiple-sets feasibility problem. For the method, we first showed that the sequences generated by the algorithm are summable, which guarantees that the distance between two adjacent iterates converges to zero, and then we established the global convergence of the algorithm provided that the penalty parameter tends to zero.
